# Neonatal Conjunctivitis Caused by *Neisseria meningitidis* US Urethritis Clade, New York, USA, August 2017

**DOI:** 10.3201/eid2505.181631

**Published:** 2019-05

**Authors:** Cecilia B. Kretz, Genevieve Bergeron, Margaret Aldrich, Danielle Bloch, Paula E. Del Rosso, Tanya A. Halse, Belinda Ostrowsky, Qinghuan Liu, Edimarlyn Gonzalez, Enoma Omoregie, Ludwin Chicaiza, Greicy Zayas, Bun Tha, Angela Liang, Jade C. Wang, Michael Levi, Scott Hughes, Kimberlee A. Musser, Don Weiss, Jennifer L. Rakeman

**Affiliations:** Centers for Disease Control and Prevention, Atlanta, Georgia, USA (C.B. Kretz, G. Bergeron);; New York City Department of Health and Mental Hygiene,; Queens, New York, USA (C.B. Kretz, G. Bergeron, M. Aldrich, D. Bloch, P.E. Del Rosso, Q. Liu, E. Gonzalez, E. Omoregie, L. Chicaiza, G. Zayas, B. Tha, A. Liang, J.C. Wang, S. Hughes, D. Weiss, J.L. Rakeman);; Montefiore Medical Center, Bronx, New York, USA (M. Aldrich, B. Ostrowsky, M. Levi);; New York State Department of Health, Albany, New York, USA (T.A. Halse, K.A. Musser)

**Keywords:** neonatal conjunctivitis, meningitis/encephalitis, whole-genome sequencing, Neisseria meningitidis, bacteria, US urethritis clade, antimicrobial resistance, men who have sex with men, MSM, New York City, New York, United States

## Abstract

We characterized a case of neonatal conjunctivitis in New York, USA, caused by *Neisseria meningitidis* by using whole-genome sequencing. The case was a rare occurrence, and the isolate obtained belonged to an emerging clade (*N. meningitidis* US nongroupable urethritis) associated with an increase in cases of urethritis since 2015.

Neonatal conjunctivitis caused by *Neisseria meningitidis* is a rare occurrence. Using whole-genome sequencing, we analyzed an isolate of *N. meningitidis* from a 3-day-old infant in New York, USA, who was given a diagnosis of unilateral conjunctivitis.

## The Study

On August 31, 2017, a 3-day-old infant was given a diagnosis of unilateral conjunctivitis caused by *Neisseria meningitidis*. The infant was born in a New York City, New York, USA, hospital to a healthy mother by cesarean section performed for prolonged rupture of membranes. A conjunctival swab specimen isolated *N. meningitidis*, which was identified by real-time PCR as nongroupable *N. meningitidis*. Nongroupable *N. meningitidis* lacks capsule production, which is a major virulence factor in causing invasive meningococcal disease. The infant received erythromycin gonococcal ophthalmic prophylaxis according to standard care guidelines and bacitracin/polymyxin ointment for empiric treatment of conjunctivitis and was discharged to home.

When *N. meningitidis* was identified on day 4 of life, the infant was hospitalized and underwent a sepsis workup that included blood and cerebrospinal fluid cultures (both showed negative results). Upon review of the limited literature available on primary meningococcal conjunctivitis, we determined that, although the conjunctivitis had improved, a 5-day course of therapy with parenteral ceftriaxone was warranted. The infant was discharged on day 8 of life.

*N. meningitidis* is a rare but known cause of conjunctivitis in newborns. A case of *N. meningitidis* perinatal transmission linked to genital and nasopharyngeal parental colonization has been described (*1*). In the case-patient described in our study, early onset of illness suggests that the infant might have acquired the infection intrapartum from maternal *N. meningitidis* vaginal colonization.

Because of concerns for parental *N. meningitidis* colonization and possible future transmission to the newborn, we offered meningococcal postexposure chemoprophylaxis to the parents. The mother received 1 dose of ciprofloxacin, which is one of the options for meningococcal postexposure chemoprophylaxis. Maternal nasopharyngeal and vaginal cultures were obtained 3 weeks after the infant’s diagnosis and maternal chemoprophylaxis, at which time the mother was able to be evaluated. Maternal cultures showed negative results. The infant’s father declined to provide screening cultures.

Whole-genome sequencing performed at the New York City Public Health Laboratory showed that the conjunctival isolate had specific molecular characteristics found in the emerging US *N. meningitidis* nongroupable urethritis clade associated with an increase in cases of urethritis in Ohio, Michigan (*2**,**3*), and other locations (*4*). This novel clade belongs to sequence type 11/clonal complex 11, a hyperinvasive lineage that has caused large meningococcal outbreaks (*5**,**6*).

Some specific characteristics found in urethritis-associated *N. meningitidis* genomes include multigene deletion at the capsule locus, causing its nongroupable phenotype, and complete acquisition of a functional *nor*B–*ani*A gene cassette, a gonococcal-acquired gene suspected to enable survival in anaerobic environments (*7*). The *ani*A gene is commonly found in *N. meningitidis*. However, many meningococcal strains have frameshift mutations in the operon.

We performed antimicrobial drug susceptibility tests for the isolate by using Etest and obtained results similar to those for other isolates of the *N. meningitidis* urethritis clade (*3*). The isolate was sensitive to azithromycin, cefotaxime, ceftriaxone, chloramphenicol, ciprofloxacin, levofloxacin, meropenem, minocycline, rifampin, and trimethoprim/sulfamethoxazole and had intermediate susceptibility to penicillin and ampicillin.

We used whole-genome–based single nucleotide polymorphism (SNP) to compare the isolate from the infant with 9 publicly available *N. meningitidis* urethritis sequences (PubMLST, https://pubmlst.org) (*4**,**8*) and 48 meningococcal sequences from New York City that belonged to sequence type 11. These sequences were from isolates obtained from invasive cases detected by surveillance: nonsterile sources, such as urethral, anal, and oropharyngeal specimens obtained during a men who have sex with men (MSM) carriage study conducted in 2016.

Genomic analyses confirmed that the conjunctivitis isolate was phylogenetically part of the *N. meningitidis* urethritis clade previously described (*2*,*4*,*7*) (Figure) because it clustered with available sequences that had only 20–50 SNP differences. This clade was composed mostly of urethral and anal isolates. However, 1 other conjunctivitis-associated isolate from 2015 that was identified from an immunosuppressed 43-year-old woman in New York City (Figure) and an oropharyngeal isolate collected during the MSM carriage study in 2016 (S. Ngai et al., pers. comm.) (Figure) were also part of the *N. meningitidis* urethritis clade. One isolate from the *N. meningitidis* urethritis clade had caused invasive meningococcal disease in a healthy, previously vaccinated, non-MSM man who had a history of childhood meningitis (Figure).

**Figure:**
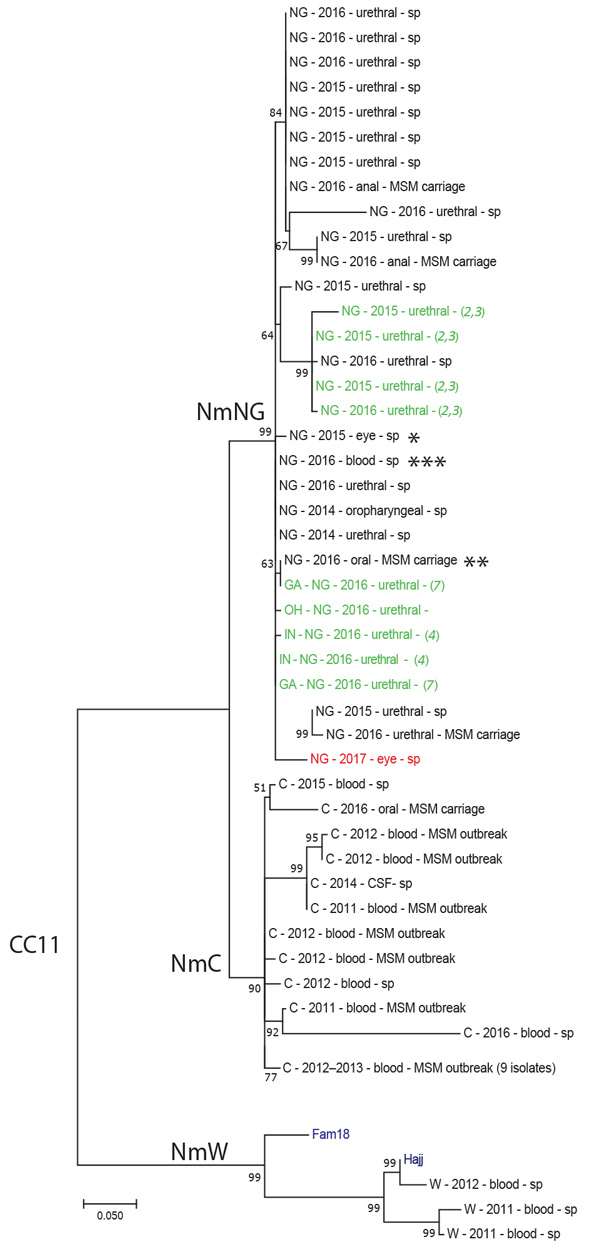
Molecular phylogenetic analysis of *Neisseria meningitidis* based on whole-genome sequence data from New York City, New York, USA, and publicly available sequences belonging to the multilocus sequence typing group cc11. Isolates are listed with serogroup, year of isolation, source, and study (sp, MSM carriage [for isolates obtained during the 2016 MSM carriage study in New York City], or reference from where they were obtained). *N. meningitidis* reference sequences are labeled in blue (Hajj and Fam18), *N. meningitidis* *Nm* urethritis clade references in green, and the 2017 conjunctivitis isolate in red. *2015 eye isolate; **oropharyngeal isolate from the MSM carriage study; ***invasive isolate from 2016. Numbers along branches are bootstrap values. The tree is drawn to scale with branch lengths measured in number of nucleotide substitutions per site. Internal nodes are labeled with bootstrap values (500 iterations). Branches are labeled with associated serogroups. Scale bar is based on 10,760 positions in the core single-nucleotide polymorphism matrix. cc, clonal complex; CSF, cerebrospinal fluid; MSM, men who have sex with men; *Nm*C, *N. meningitidis* serogroup C; *Nm*W, *N. meningitidis* serogroup W; *Nm*NG, *N. meningitidis* nongroupable; sp, sporadic case.

These findings are unusual because only a few invasive isolates have been described as part of the *N. meningitidis* US nongroupable urethritis clade (*8*). These observations demonstrate that strains belonging to this clade can cause invasive disease. However, additional information on these cases to characterize risk factors is limited. Although surveillance of *N. meningitidis* is primarily focused on invasive disease, *N. meningitidis* has been reported from noninvasive sites, such as urethral and oropharyngeal sites. In addition, few cases of primary meningococcal conjunctivitis are reported in the literature.

## Conclusions

Our study showed that an *N. meningitidis* isolate belonging to the sequence type 11/clonal complex 11 clade with multigene deletion at the capsule locus has likely caused neonatal conjunctivitis in this child. Strains from this clade have the genetic potential to cause invasive meningococcal disease. The organism was not recovered from the infant’s mother. However, she was not tested until 3 weeks after identification of the *N. meningitidis* isolate and after postexposure prophylaxis. The infant’s infection might have been healthcare associated, but was more likely acquired by maternal genitourinary colonization ascending to the amniotic fluid during the period of prolonged rupture of membranes.

Modes of transmission are not fully understood for the *N. meningitidis* US nongroupable urethritis clade. Given its potential to cause invasive disease, more studies are needed to increase our understanding and to inform control and prevention measures. A better understanding of *N. meningitidis* noninvasive disease, including conjunctivitis in newborns and urethritis, will improve our understanding of the clinical manifestation, transmission modes, and epidemiologic evolution of *N. meningitidis* strains.
